# Multi-Omics Integration Reveals Mitochondrial Gene Regulation as a Determinant of Tuberculosis Susceptibility: A Mendelian Randomization Approach

**DOI:** 10.3390/biomedicines13030749

**Published:** 2025-03-19

**Authors:** Tingting Fang, Yu Chen, Feifei Yuan, Yuyan Ma, Qingqing Wang, Yumeng Yao, Sishi Cai, Wenting Jin, Qing Miao, Bijie Hu

**Affiliations:** Department of Infectious Diseases, Zhongshan Hospital, Fudan University, 180 Fenglin Road, Shanghai 200032, China; fang.tingting@zs-hospital.sh.cn (T.F.); chenyuysfd@163.com (Y.C.); yuan.feifei@zs-hospital.sh.cn (F.Y.); ma.yuyan@zs-hospital.sh.cn (Y.M.); wang.qingqing@zs-hospital.sh.cn (Q.W.); yao.yumeng@zs-hospital.sh.cn (Y.Y.); cai.sishi@zs-hospital.sh.cn (S.C.); jin.wenting@zs-hospital.sh.cn (W.J.)

**Keywords:** tuberculosis susceptibility, mitochondrial gene regulation, multi-omics integration, Mendelian randomization, immune response, biomarkers

## Abstract

**Background/Objectives:** Mitochondrial dysfunction has been implicated in the pathogenesis of tuberculosis (TB). Despite emerging evidence of the importance of mitochondrial gene regulation in the immune response, the specific role of mitochondrial-related genes in TB susceptibility remains to be fully elucidated. **Methods:** We employed a multi-omics approach integrating genetic, methylation, and protein-level data. Mendelian randomization (MR) and colocalization analyses were conducted to explore causal associations between mitochondrial gene features—expression quantitative trait loci (eQTL), methylation quantitative trait loci (mQTL), and protein quantitative trait loci (pQTL)—and TB susceptibility. Data were obtained from the FinnGen cohort and validated using independent datasets. **Results:** Our analyses identified several key mitochondrial genes (e.g., ACSF3, AK3, LYRM4, and PDHB) significantly associated with TB susceptibility. Random forest analysis and gene set enrichment analysis (GSEA) supported the predictive power of these genes. Furthermore, we observed significant correlations between mitochondrial gene expression and immune cell infiltration in TB patients, suggesting a role of these genes in modulating immune responses during infection. Receiver operating characteristic (ROC) analysis confirmed strong predictive accuracy for the identified feature genes, with area under the curve (AUC) values exceeding 0.7. **Conclusions:** This study demonstrates that mitochondrial-related gene regulation influences TB susceptibility across genetic, methylation, and protein levels. The integration of multi-omics data provides valuable insight into the molecular mechanisms underlying TB, highlighting the potential of mitochondrial genes as biomarkers and therapeutic targets.

## 1. Introduction

Tuberculosis (TB) remains a leading cause of morbidity and mortality worldwide, despite the availability of a vaccine for over a century and effective treatments for more than 75 years [1]. According to the World Health Organization (WHO), approximately 10 million people develop TB annually, and over 1.4 million succumb to the disease, highlighting the urgent need for improved prevention and treatment strategies [2]. Despite the availability of effective anti-TB treatments, the rise of drug-resistant strains, prolonged therapy regimens, and patient non-adherence continue to hinder global TB control efforts [2]. Mycobacterium tuberculosis (Mtb), the causative agent of TB, is highly adept at evading host immune defenses, persisting within macrophages and complicating effective immune clearance [3,4]. While advances have been made in understanding the host–pathogen interactions that contribute to TB pathogenesis, much remains to be understood, particularly regarding the host factors that influence susceptibility to Mycobacterium tuberculosis infection.

Mitochondria, as central regulators of energy metabolism, are crucial to the host immune response [5]. Beyond ATP production, they regulate immune cell activation, apoptosis, and inflammatory responses, linking cellular metabolism with immune function [6]. Dysfunctional mitochondria can compromise immune cell performance, weakening the host’s defense mechanisms against infectious diseases, including TB [7,8,9].

Emerging evidence highlights mitochondrial dysfunction’s role in infectious and inflammatory conditions [7,8,9,10,11]. Proper mitochondrial regulation is essential for immune cells like macrophages and T cells, which are key in combating M. tuberculosis. Dysfunction may impair immune regulation, affecting the host’s ability to control TB infection [12]. However, the genetic basis of mitochondrial dysfunction in TB susceptibility remains poorly understood, particularly when considering a comprehensive multi-omics approach that integrates genetic, epigenetic, and proteomic data.

In this study, we sought to unravel the complex relationship between mitochondrial gene regulation and TB susceptibility by employing an integrative multi-omics approach. We utilized Mendelian randomization (MR) and colocalization analyses to assess the causal effects of mitochondrial gene expression, methylation, and protein abundance on TB susceptibility. Our study aimed to identify key mitochondrial genes and pathways involved in TB pathogenesis, providing a deeper understanding of mitochondrial dysfunction in the context of TB and identifying potential biomarkers for early detection and intervention.

## 2. Materials and Methods

**Figure 1:**
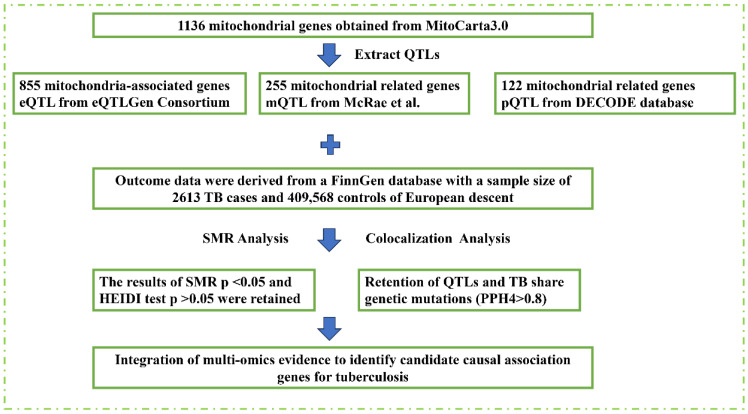
Study design. Overview of the study design for identifying candidate causal genes for tuberculosis (TB) susceptibility. QTL, quantitative trait loci; eQTL, expression QTL; mQTL, methylation QTL; pQTL, protein QTL; SMR, summary-based Mendelian randomization; HEIDI, heterogeneity in dependent instruments; PPH4, posterior probability of H4.

This study employed an integrative multi-omics approach to investigate the association between mitochondrial gene regulation and tuberculosis (TB) susceptibility (Figure 1). Mitochondrial gene information was sourced from the MitoCarta 3.0 database [13]. We used various publicly available datasets, including genetic, methylation, and protein-level data, to comprehensively examine the role of mitochondrial gene regulation in TB.

### 2.1. Data Sources and Quality Control

Expression quantitative trait loci (eQTL) data were obtained from the eQTLGen Consortium [14] (31,684 blood and PBMC samples). Methylation quantitative trait loci (mQTL) data were sourced from the Brisbane Systems Genetics Study and Lothian Birth Cohorts [15] (1980 participants). Protein quantitative trait loci (pQTL) data were downloaded from the DECODE database [16], covering 4907 aptamers from 35,559 individuals. Summary statistics for TB susceptibility were obtained from the FinnGen cohort (2613 TB cases, 409,568 controls). Tissue-specific gene expression data were validated using the GTEx project. RNA-seq data from TB patients and healthy controls (GSE193777, GSE249575) [17] were obtained from the Gene Expression Omnibus (GEO) (Table S1). All datasets underwent uniform quality control, including linkage disequilibrium (LD) pruning, minor allele frequency (MAF) filtering (>0.02), and Hardy–Weinberg equilibrium testing.

### 2.2. Mendelian Randomization and Colocalization Analysis

A two-stage Mendelian randomization (MR) approach was used to determine the causal relationship between mitochondrial gene regulation and TB susceptibility. Genetic variants associated with mitochondrial gene function, including expression, methylation, and protein abundance, were selected as instrumental variables (IVs). The summary-data-based MR (SMR) analysis [18] was employed to identify pleiotropic associations, followed by the HEIDI test to differentiate true pleiotropy from linkage (*p* > 0.05 indicating absence of linkage) [19]. Colocalization analysis was performed using the coloc R package (version: 5.2.3, https://github.com/chr1swallace/coloc, accessed on 30 January 2025) [20] to determine whether genetic signals influenced both gene regulation and TB susceptibility (PP.H4 > 0.8 indicating colocalization).

### 2.3. Differential Expression and Functional Enrichment Analysis

Differential expression analysis was conducted using the limma R package [21] on RNA-seq data from TB patients and healthy controls. Genes with |LogFC| > 0.25 and *p* < 0.05 were considered significantly differentially expressed. Overlapping differentially expressed genes (DEGs) with MR-identified candidate causal genes were analyzed using the STRING database [22] to construct a protein–protein interaction (PPI) network visualized with Cytoscape (version 3.9.1, https://cytoscape.org/, accessed on 30 January 2025) [23]. Gene ontology (GO) terms and KEGG pathways associated with candidate genes were analyzed using the clusterProfiler R package (version 4.10.1, https://bioconductor.org/packages/release/bioc/html/clusterProfiler.html, accessed on 30 January 2025) [24].

Gene set enrichment analysis (GSEA) was performed to evaluate whether specific gene sets were enriched between high- and low-expression groups of the identified model genes. Pathway enrichment analysis was conducted using clusterProfiler (version 4.10.1, https://bioconductor.org/packages/release/bioc/html/clusterProfiler.html, accessed on 30 January 2025) with MSigDB (version 4.3.0, https://www.gsea-msigdb.org/gsea/msigdb/index.jsp, accessed on 14 October 2024) background datasets, including KEGG and reactome pathways. Enrichment results were visualized using the gseaplot2 function, with a significance threshold of *p* < 0.05.

### 2.4. Feature Gene Analysis and Validation

Feature gene identification was carried out using random forest analysis with the randomForest R package (version 4.7-1.1, https://cran.r-project.org/web/packages/randomForest/) [25]. Genes with a mean decrease accuracy (MDA) score greater than 1 were selected for further analysis. These feature genes were validated using the Wilcoxon rank-sum test in the independent GSE34608 dataset, comparing TB patients and healthy controls. Boxplots were generated using ggplot2 (version 3.5.0, https://cran.r-project.org/web/packages/ggplot2/index.html, accessed on 16 October 2024) to visualize gene expression differences. The diagnostic accuracy of feature genes was evaluated using receiver operating characteristic (ROC) curves generated with the pROC R package (version 1.18.56, https://cran.r-project.org/web/packages/pROC/index.html, accessed on 24 October 2024) [26].

### 2.5. Immune Cell Infiltration Analysis

Immune cell infiltration was evaluated using single-sample gene set enrichment analysis (ssGSEA) with the GSVA R package (version 1.50.5, https://www.bioconductor.org/packages/release/bioc/html/GSVA.html, accessed on 1 November 2024). Gene sets for immune cell markers, including 28 immune cell types as provided by Bindea et al. [27], were used to calculate enrichment scores. Differences in immune cell infiltration between TB and control samples were evaluated using the Wilcoxon rank-sum test.

To explore the correlation between mitochondrial gene expression and immune cell infiltration, Pearson correlation analysis was performed using the corrplot R package (version 0.92, https://github.com/taiyun/corrplot, accessed on 4 November 2024). Correlations were considered significant at *p* < 0.05 and |r| > 0.5, and heatmaps were generated to visualize these associations.

## 3. Results

### 3.1. Genetic, Epigenetic, and Protein-Level Associations of Mitochondrial Genes with TB Susceptibility

**Figure 2:**
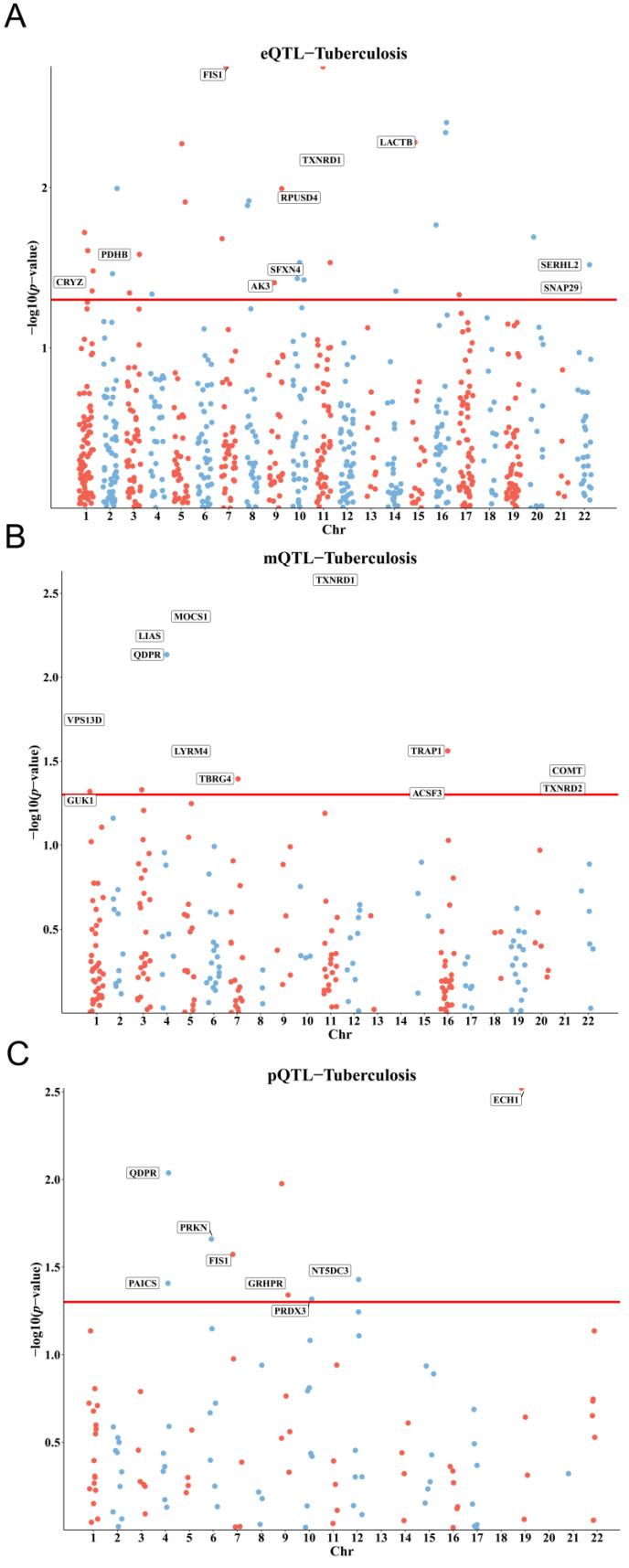
Manhattan plots for mitochondrial-related gene expression (**A**), methylation (**B**), and protein abundance (**C**), highlighting significant genetic loci linked to TB susceptibility. The red and blue dots in these Manhattan plots represent the direction of the effect of the genetic variants on the respective molecular traits: red dots indicate variants with a positive effect and blue dots indicate variants with a negative effect. red horizontal lines in each subfigure indicate the threshold for statistical significance. Points above these lines represent genetic variants significantly associated with the respective molecular traits.

This study identified significant associations between mitochondrial gene regulation and tuberculosis (TB) susceptibility at genetic, epigenetic, and protein levels using eQTL, mQTL, and pQTL data. Specifically, out of 855 mitochondrial eQTLs analyzed, 33 showed significant associations with TB risk, with 10 maintaining causal relationships after excluding heterogeneity. For mQTLs, 15 out of 255 loci showed significant associations, and 12 were confirmed as causal. The analysis of 122 mitochondria-related pQTLs identified nine loci significantly associated with TB, with eight retaining significance post-heterogeneity analysis (Table S2). Together, these results highlight the consistent involvement of mitochondrial gene regulation in TB susceptibility across various molecular layers (Figure 2A–C).

### 3.2. Colocalization and Integration of Multi-Omics Evidence for Causal Genes

**Figure 3:**
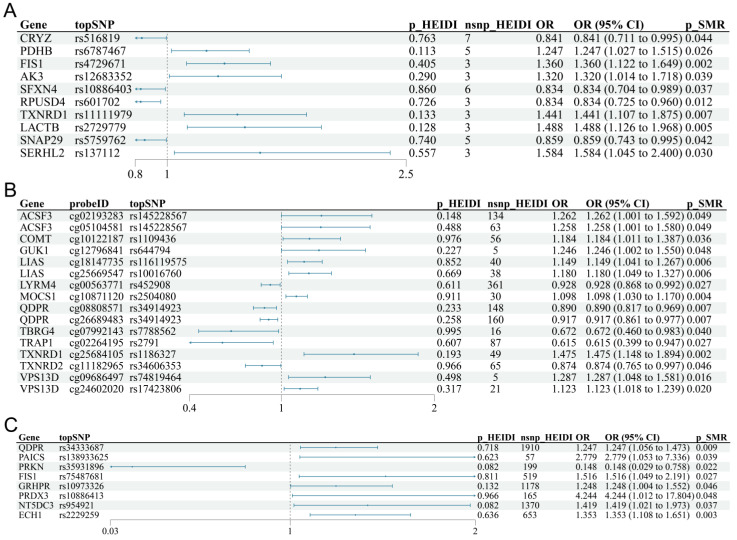
Forest plots showing causal associations for mitochondrial gene regulation with tuberculosis (TB) susceptibility across three levels after heterogeneity exclusion: (**A**) eQTL, (**B**) mQTL, and (**C**) pQTL. OR, odds ratio; CI, confidence interval. Each horizontal line represents the 95% confidence interval (CI) of the odds ratio (OR) for the genetic variant’s causal association with tuberculosis susceptibility. The vertical dashed line indicates the null effect (OR = 1). If the horizontal line crosses the vertical dashed line, the association is not statistically significant; lines entirely on one side indicate statistically significant associations.

Colocalization analysis using eQTL, mQTL, and pQTL data showed no evidence of shared causal variants with TB susceptibility, as indicated by posterior probability hypothesis 4 (PPH4) values below 0.8 for all analyzed loci (Table S3). Integration across multi-omics layers identified no genes fulfilling the criteria as primary causal factors. Notably, QDPR exhibited protective effects at the methylation level but posed risks at the protein level; however, it lacked adequate eQTL support. FIS1 showed risk effects in eQTL and pQTL analyses but lacked sufficient mQTL data (Table 1). These results suggest independent genetic effects across omics layers and emphasize the complex landscape of gene regulation (Figure 3A–C).

### 3.3. Differential Expression Analysis and Identification of Key Mitochondrial Genes

**Figure 4:**
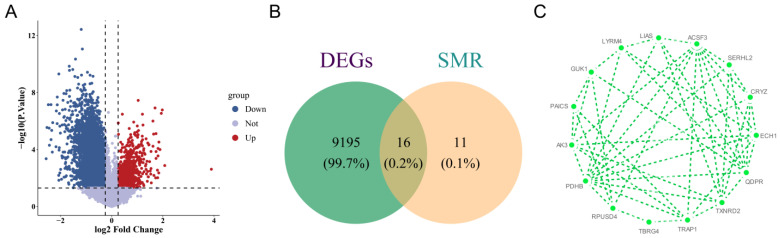
Integrated plots showing mitochondrial gene involvement in tuberculosis (TB): (**A**) volcano plot of differentially expressed genes (DEGs) between TB patients and healthy controls, (**B**) Venn diagram of overlapping DEGs and MR-identified causal genes, and (**C**) protein–protein interaction (PPI) network of 16 overlapping genes. The vertical dashed lines represent the threshold values of log2 fold-change (±1), distinguishing significantly upregulated (red) and downregulated (blue) genes. The horizontal dashed line indicates the statistical significance threshold (P-value cutoff), above which genes are considered significantly differentially expressed.

Differential expression analysis revealed 9211 significantly differentially expressed genes (DEGs) between TB patients and healthy controls, with 901 upregulated and 8310 downregulated genes. We intersected these DEGs with candidate causal genes from MR and QTL analyses, resulting in 16 overlapping genes with strong evidence of involvement in TB susceptibility. These overlapping genes were considered key candidates for their roles in TB pathogenesis, supported by both differential expression and genetic causality evidence (Figure 4A–C).

### 3.4. Functional Enrichment of Overlapping Genes

**Figure 5:**
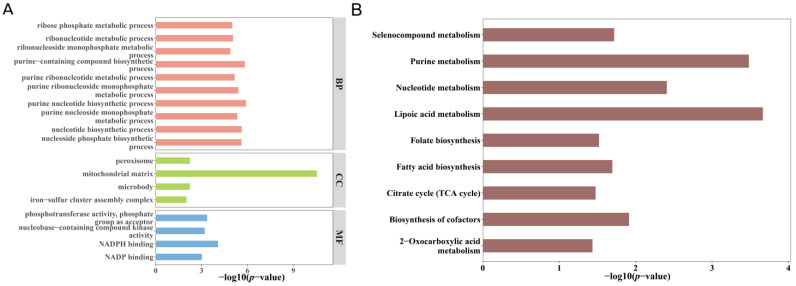
Enrichment analysis of overlapping genes involved in tuberculosis (TB) susceptibility indicating key biological processes and pathways linked to mitochondrial gene regulation in TB: (**A**) top 10 enriched gene ontology (GO) terms and (**B**) enriched KEGG pathways.

The functional enrichment analysis revealed significant enrichment of the 16 overlapping genes in processes related to oxidative phosphorylation and immune regulation, suggesting that mitochondrial dysfunction plays a pivotal role in TB pathogenesis (Table 2). The enrichment was observed in 94 GO biological processes, four cellular components, four molecular functions, and nine KEGG pathways (Figure 5A,B).

### 3.5. Identification and Validation of Key Predictive Genes

**Figure 6:**
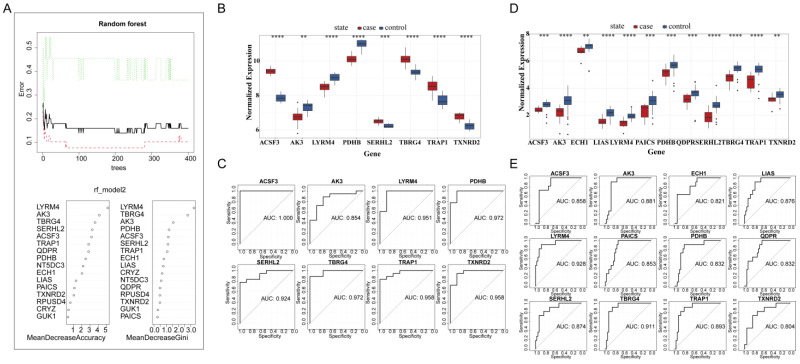
Identification and validation of key predictive genes for TB susceptibility demonstrating their predictive accuracy in identifying tuberculosis (TB) susceptibility: (**A**) importance scores of key predictive genes from random forest analysis, (**B**) gene expression levels in the validation set, (**C**) ROC analysis for model genes, (**D**) Wilcoxon rank-sum test results for gene expression, and (**E**) ROC curves for ACSF3, AK3, LYRM4, and PDHB. In panel (**A**), the different colored lines represent the error rates obtained from the random forest model: the black line indicates the overall classification error rate, the green line indicates the error rate for the tuberculosis (TB) patient group, and the red dashed line indicates the error rate for the healthy control group. The stabilization of these error rates signifies model convergence (** *p* < 0.01, *** *p* < 0.001, **** *p* < 0.0001).

Using random forest analysis, ACSF3, AK3, LYRM4, and PDHB were identified as key predictive genes for TB susceptibility. These genes were validated in independent datasets, demonstrating consistent expression patterns across both training and validation sets. The diagnostic accuracy was confirmed by ROC analysis with area under the curve (AUC) values exceeding 0.7 for each gene (Figure 6A–E).

### 3.6. Correlation Between Mitochondrial Gene Expression and Immune Cell Infiltration

**Figure 7:**
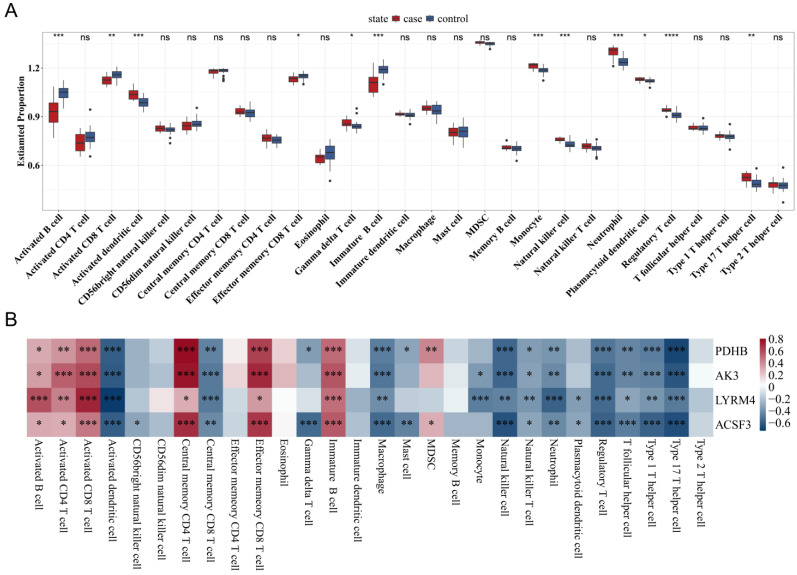
Immune cell infiltration analysis related to mitochondrial gene expression in TB highlighting the role of immune dynamics in TB pathogenesis: (**A**) immune cell infiltration levels in tuberculosis (TB) patients versus healthy controls and (**B**) significant correlations between immune cell types and the expression of key mitochondrial genes (* *p* < 0.05, ** *p* < 0.01, *** *p* < 0.001, **** *p* < 0.0001).

Single-sample gene set enrichment analysis (ssGSEA) showed significant correlations between the expression of model mitochondrial genes and immune cell infiltration levels, particularly involving macrophages and T cells, suggesting their role in modulating immune dynamics in TB (Figure 7A,B).

### 3.7. Pathway Enrichment Analysis of Mitochondrial Gene Expression

**Figure 8:**
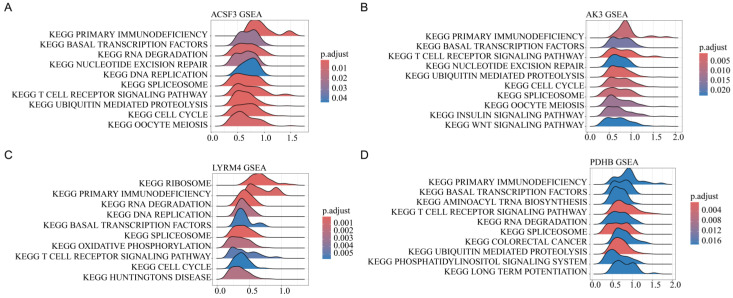
GSEA results indicating pathways enriched for ACSF3 (**A**), AK3 (**B**), LYRM4 (**C**), and PDHB (**D**) expression. The x-axis represents the normalized enrichment score (NES), with positive values indicating pathway activation in high-expression groups. Color scales correspond to adjusted *p*-values.

To elucidate the functional implications of mitochondrial gene expression, GSEA was performed to identify pathways enriched in the high and low expression of model genes. Pathways linked to oxidative phosphorylation and immune regulation were significantly enriched for ACSF3, AK3, LYRM4, and PDHB, supporting their functional involvement in TB pathogenesis (Figure 8).

## 4. Discussion

This study provides novel insights into the role of mitochondrial gene regulation in tuberculosis (TB) susceptibility through an integrative multi-omics approach. Our findings highlight the critical involvement of mitochondrial dysfunction in TB pathogenesis, emphasizing mitochondrial regulation as a significant contributor to host susceptibility to Mycobacterium tuberculosis. By combining Mendelian randomization (MR), colocalization, differential expression analysis, and protein–protein interaction (PPI) network analysis, we provide robust evidence of the involvement of mitochondrial genes in TB susceptibility, supporting the growing body of literature that links mitochondrial health with infectious disease outcomes [7,28].

The MR analysis revealed significant associations between mitochondrial gene expression and TB risk, consistent with recent studies highlighting mitochondrial–immune crosstalk in infectious diseases [7,29,30] and multi-omics approaches in TB research [31,32]. However, colocalization analysis indicated no strong evidence of shared causal variants, implying that the observed associations may arise from independent genetic effects rather than pleiotropic influences. This finding is consistent with those of previous studies, which suggest that mitochondrial dysfunction can exacerbate infection susceptibility through distinct mechanisms not necessarily overlapping with TB risk loci. For instance, studies have shown that mitochondrial dysfunction in immune cells can contribute to increased inflammation, which, in turn, may enhance the risk of chronic infections like TB [7,12]. These results support the hypothesis that mitochondrial regulation is more focused on immune dysregulation and energy metabolism, which could increase host vulnerability to TB.

Differential expression analysis identified 16 mitochondrial genes as key candidates involved in TB pathogenesis. Feature genes such as ACSF3, AK3, LYRM4, and PDHB were found to be highly predictive of TB susceptibility, indicating their essential roles in metabolic regulation and immune response modulation. For example, ACSF3 is involved in mitochondrial fatty acid metabolism, critical for maintaining energy balance during immune activation [33,34]. Elevated ACSF3 expression may reflect an increased requirement for β-oxidation in activated immune cells, highlighting the significance of energy metabolism in host defense against M. tuberculosis. Similarly, AK3’s involvement in cellular energy homeostasis and LYRM4’s role in mitochondrial respiratory chain complex assembly underline their importance in efficient immune function, whereas PDHB links glycolysis to the tricarboxylic acid (TCA) cycle, underscoring its role in metabolic flexibility during infection [35,36,37,38]. Collectively, these findings emphasize that mitochondrial genes contribute to TB susceptibility by modulating metabolic pathways crucial for effective immune cell function.

The correlation between mitochondrial gene expression and immune cell infiltration provides important insights into the influence of mitochondrial dysfunction on TB immune dynamics. Our study demonstrated significant correlations between key feature genes (ACSF3, AK3, LYRM4, and PDHB) and immune cell infiltration, particularly macrophages and T cells, which are pivotal in TB control. Macrophages, being primary host cells for M. tuberculosis, rely heavily on mitochondrial function for phagocytosis and bacterial clearance. The observed correlation between mitochondrial dysfunction and macrophage infiltration suggests that impaired mitochondrial function may compromise macrophage-mediated immune responses, thereby increasing TB susceptibility. This highlights the potential role of mitochondrial regulation in influencing the immune microenvironment, ultimately affecting the host’s ability to control TB infection. Studies have shown that macrophages with compromised mitochondrial function exhibit reduced pathogen clearance and increased inflammation, which are central features of TB pathology [39,40].

The GSEA results in Figure 8 reveal the significant enrichment of immune-related pathways, such as primary immunodeficiency and T cell receptor signaling, across ACSF3, AK3, LYRM4, and PDHB, suggesting their roles in modulating TB susceptibility via immune dysregulation. Additionally, the enrichment of cell cycle pathways indicates that these mitochondrial genes may influence TB pathogenesis by affecting host cell proliferation. The identification of mitochondrial genes associated with TB susceptibility has several clinical implications. These genes could serve as potential biomarkers for early diagnosis, aiding in the identification of individuals at a higher risk for TB. Furthermore, therapeutic interventions targeting mitochondrial function—such as enhancing oxidative phosphorylation or stabilizing mitochondrial dynamics—could be promising strategies to improve host resistance against TB, especially in individuals with mitochondrial dysfunction. Our study provides a theoretical foundation for the potential of mitochondrial-targeted therapies as adjuncts to conventional TB treatment, aiming to improve treatment efficacy and reduce TB burden in the future. This is particularly important as multidrug resistant TB (MDR-TB) becomes more prevalent, and new therapeutic strategies are urgently needed.

One key limitation of this study is the lack of diversity in the datasets used. Most data were derived from individuals of European ancestry, which may limit the generalizability of our findings to other populations. Genetic and environmental factors influencing TB susceptibility vary significantly across different ethnic groups, and our results may not be directly applicable to non-European populations. As demonstrated by previous research, the genetic architecture of TB susceptibility can differ substantially across populations [41]. Future studies should aim to validate these findings in more ethnically diverse cohorts and explore whether the identified mitochondrial associations hold across different genetic backgrounds. Additionally, the complex interplay between mitochondrial regulation and host immunity warrants deeper investigation using advanced approaches, such as single-cell multi-omics or longitudinal studies, to capture temporal dynamics during TB infection. Expanding the scope to include diverse populations and sophisticated methodologies will help enhance the applicability of our findings in global TB control efforts.

In conclusion, this study underscores the significant role of mitochondrial gene regulation in TB susceptibility. Through comprehensive integration of genetic, epigenetic, and transcriptomic data, we identified pivotal mitochondrial genes that contribute to TB pathogenesis. Our findings provide a foundation for developing novel diagnostic and therapeutic strategies targeting mitochondrial health, ultimately aiming to improve outcomes for individuals at risk of TB. Future research should focus on the clinical validation of these biomarkers and exploration of mitochondrial-targeted therapies to enhance TB treatment efficacy and mitigate the disease burden.

## 5. Conclusions

In conclusion, this study identifies mitochondrial gene regulation as a key factor in TB susceptibility. Through an integrative multi-omics approach, we highlight key genes such as ACSF3, AK3, LYRM4, and PDHB, which influence TB risk by modulating immune responses and oxidative phosphorylation. Our use of Mendelian randomization provides robust evidence for the causal role of these genes, suggesting mitochondrial dysfunction as an independent contributor to TB susceptibility. These findings offer new opportunities for mitochondrial-targeted therapies and biomarkers, advancing both TB diagnostics and treatment strategies.

## Figures and Tables

**Table 1:** Summary of integrated multi-omics evidence for candidate causal genes.

Gene	pQTL			mQTL				eQTL		
	p_SMR	p_HEIDI	OR (95% CI)	p_SMR	p_HEIDI	probeID	OR (95% CI)	p_SMR	p_HEIDI	OR (95% CI)
QDPR	0.00917	0.718	1.2473 (1.0563 to 1.4729)	0.0073	0.2330	cg08808571	0.8901 (0.8174 to 0.9692)			
				0.0071	0.2580	cg26689483	0.9173 (0.8614 to 0.9768)			
PAICS	0.0391	0.623	2.7788 (1.0526 to 7.3362)							
PRKN	0.0219	0.082	0.1479 (0.0289 to 0.7579)							
FIS1	0.0267	0.811	1.5163 (1.0491 to 2.1914)					0.0018	0.4049	1.3601 (1.1218 to 1.6489)
GRHPR	0.0456	0.132	1.2484 (1.0043 to 1.5519)							
PRDX3	0.0482	0.966	4.2439 (1.0116 to 17.8040)							
NT5DC3	0.0372	0.082	1.4193 (1.0209 to 1.9731)							
ECH1	0.0030	0.636	1.3526 (1.1079 to 1.6512)							
ACSF3				0.0490	0.1480	cg02193283	1.2622 (1.0010 to 1.5916)			
ACSF3				0.0490	0.4880	cg05104581	1.2576 (1.0010 to 1.5800)			
COMT				0.0359	0.9760	cg10122187	1.1841 (1.0111 to 1.3866)			
GUK1				0.0479	0.2270	cg12796841	1.2462 (1.0021 to 1.5497)			
LIAS				0.0057	0.8520	cg18147735	1.1486 (1.0412 to 1.2670)			
LIAS				0.0059	0.6690	cg25669547	1.1797 (1.0487 to 1.3271)			
LYRM4				0.0275	0.6110	cg00563771	0.9275 (0.8675 to 0.9917)			
MOCS1				0.0043	0.9110	cg10871120	1.0976 (1.0296 to 1.1702)			
TBRG4				0.0403	0.9950	cg07992143	0.6720 (0.4596 to 0.9826)			
TRAP1				0.0275	0.6070	cg02264195	0.6152 (0.3994 to 0.9475)			
TXNRD1				0.0023	0.1930	cg25684105	1.4748 (1.1485 to 1.8938)			
TXNRD2				0.0457	0.9660	cg11182965	0.8736 (0.7652 to 0.9974)			
VPS13D				0.0159	0.4980	cg09686497	1.2873 (1.0484 to 1.5806)			
VPS13D				0.0201	0.3170	cg24602020	1.1231 (1.0183 to 1.2386)			
CRYZ								0.0441	0.7625	0.8411 (0.7107 to 0.9954)
PDHB								0.0261	0.1126	1.2471 (1.0266 to 1.5149)
AK3								0.0392	0.2904	1.3196 (1.0139 to 1.7176)
SFXN4								0.0369	0.8596	0.8345 (0.7041 to 0.9890)
RPUSD4								0.0115	0.7258	0.8342 (0.7248 to 0.9602)
TXNRD1								0.0067	0.1326	1.4405 (1.1066 to 1.8751)
LACTB								0.0052	0.1275	1.4885 (1.1261 to 1.9676)
SNAP29								0.0421	0.7399	0.8594 (0.7427 to 0.9946)
SERHL2								0.0303	0.5571	1.5837 (1.0448 to 2.4005)
QTL, quantitative trait loci										

**Table 2:** Logistic regression analysis of 16 overlapping genes with TB risk.

Gene	*p*-Value	OR	OR (95% CI)
SERHL2	0.001	0.0682	0.0682 (0.0138 to 0.3369)
TBRG4	0.0013	0.0449	0.0449 (0.0068 to 0.2979)
LIAS	0.0014	0.0149	0.0149 (0.0011 to 0.1978)
NT5DC3	0.0021	0.0025	0.0025 (0.0001 to 0.1145)
TRAP1	0.0023	0.1349	0.1349 (0.0372 to 0.4891)
LYRM4	0.0027	1.00 × 10^−4^	0.0001 (0.0000 to 0.0381)
PAICS	0.0033	0.1412	0.1412 (0.0383 to 0.5201)
AK3	0.0045	0.2057	0.2057 (0.0692 to 0.6117)
TXNRD2	0.0122	0.0488	0.0488 (0.0046 to 0.5178)
ACSF3	0.0134	0.0546	0.0546 (0.0055 to 0.5471)
PDHB	0.0136	0.2084	0.2084 (0.0600 to 0.7245)
RPUSD4	0.0157	0.2447	0.2447 (0.0781 to 0.7671)
GUK1	0.0158	3.45	3.4500 (1.2623 to 9.4289)
CRYZ	0.0191	0.3339	0.3339 (0.1334 to 0.8359)
QDPR	0.0431	0.2739	0.2739 (0.0781 to 0.9603)
ECH1	0.0681	0.2731	0.2731 (0.0677 to 1.1014)

## Data Availability

The sources of the data utilized in this study are available in materials and methods.

## Supplementary Materials

The following supporting information can be downloaded at: https://www.mdpi.com/article/10.3390/biomedicines13030749/s1, Figure S1: Forest plot of logistic regression results showing significant gene associations with TB risk. Figure S2: Forest plot illustrating tissue-specific validation outcomes for mitochondrial feature genes. Table S1: NCBI GEO dataset information. Table S2: Summary of eQTL, mQTL, and pQTL associations with TB. Table S3: Summary of colocalization results for eQTL, mQTL, and pQTL. Table S4: Tissue-specific validation of model genes using GTEx data.
