# Supplementary material for: Multi-Omics Integration Reveals Mitochondrial Gene Regulation as a Determinant of Tuberculosis Susceptibility: A Mendelian Randomization Approach
Source: Biomedicines. 2025 Mar 19;13(3):749. doi: 10.3390/biomedicines13030749 (PMC11939929; doi:10.3390/biomedicines13030749)
Supplement: Supplementary file 1 [file biomedicines-13-00749-s001.zip › biomedicines-3480721-supplementary.pdf]

Supplementary Materials Methods

RNA-seq data source

RNA-seq data from TB patients and healthy controls (GSE193777, GSE249575)[17] were obtained from the Gene Expression Omnibus (GEO) (Table S1).

Table S1 NCBI GEO dataset information.

Accession ID	TB	Control	Group	Platform
GSE193777	11	39	Training	Illumina HiSeq 4000
GSE249575	65	67	Test	Illumina HumanHT-12 V4.0 expression beadchip

Single-Gene Logistic Regression Analysis

Single-gene analysis was conducted using logistic regression to assess the relationship between the expression levels of mitochondrial genes and TB risk. Gene expression data were treated as the independent variable, and TB status (case/control) was used as the outcome variable. Logistic regression models were built using the glm function in R, with statistical significance set at $p < 0.05$. The results were summarized in terms of odds ratios (ORs) and corresponding 95% confidence intervals (CIs) to determine the contribution of each gene to TB susceptibility.

Tissue-Specific Validation of Mitochondrial Feature Genes

To determine the tissue-specific involvement of identified feature genes, expression levels were validated using the GTEx dataset (https://gtexportal.org/home/). Expression data for key mitochondrial genes (ACSF3, AK3, LYRM4, and PDHB) were extracted across multiple tissue types. The validation process involved calculating mean expression levels and generating heatmaps to visualize tissue-specific expression patterns. These analyses were conducted using the limma and ggplot2 R packages. The tissue-specificity of the feature genes provides insights into whether mitochondrial gene expression is predominantly regulated in lung tissues, which are directly involved in TB pathogenesis.

Supplementary results

Associations Between Mitochondrial Gene Expression and TB Risk

We used logistic regression to assess the association between the expression of the 16 overlapping genes and TB risk, revealing that 15 genes were significantly associated with TB, with 14 demonstrating protective associations and 1 presenting an increased risk (Figure S1). This analysis highlights the significant role of these genes in modulating TB susceptibility.

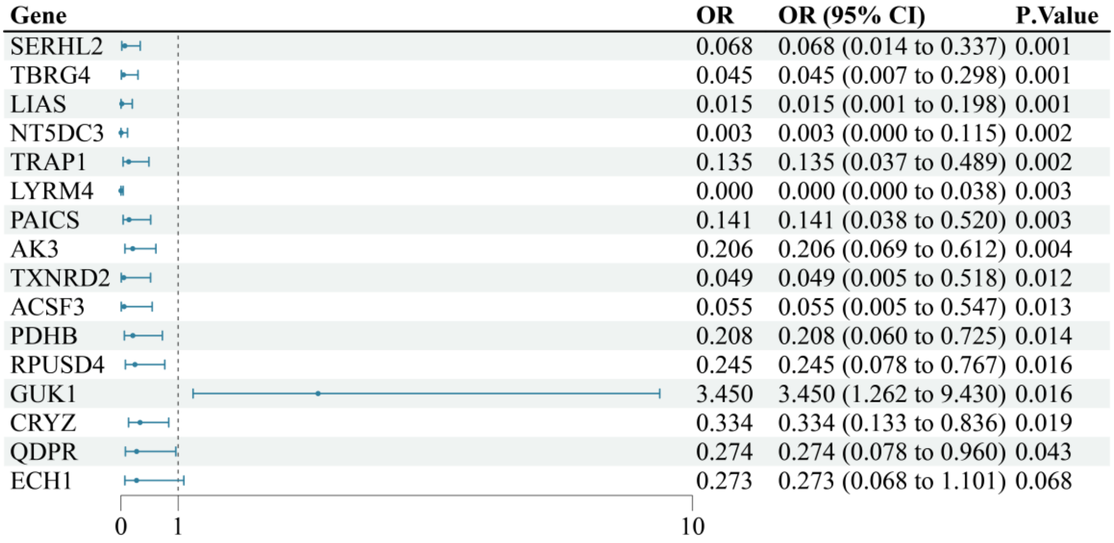

Figure S1: Forest plot of logistic regression results showing significant gene associations with TB risk.

Summary of eQTL, mQTL, and pQTL associations with TB

Analysis of 122 mitochondria-related pQTLs identified 9 loci significantly associated with TB, with 8 retaining significance post-heterogeneity testing (Table S2).

Table S2. Summary of eQTL, mQTL, and pQTL associations with TB

eQTL					mQTL						pQTL				
Chr	Gene	SNP	p_SMR	p_HEIDI	Chr	Gene	SNP	p_SMR	p_HEIDI	probeID	Chr	Gene	SNP	p_SMR	p_HEIDI
1	CRYZ	rs516819	0.0441	0.7625	16	ACSF3	rs145228567	0.0490	0.148	cg02193283	4	QDPR	rs34333687	0.0092	0.7180
3	PDHB	rs6787467	0.0261	0.1126	16	ACSF3	rs145228567	0.0490	0.488	cg05104581	4	PAICS	rs138933625	0.0391	0.6230
7	FIS1	rs4729671	0.0018	0.4049	22	COMT	rs1109436	0.0359	0.976	cg10122187	6	PRKN	rs35931896	0.0219	0.0820
9	AK3	rs12683352	0.0392	0.2904	1	GUK1	rs644794	0.0479	0.227	cg12796841	7	FIS1	rs75487681	0.0267	0.8110
10	SFXN4	rs10886403	0.0369	0.8596	4	LIAS	rs116119575	0.0057	0.852	cg18147735	9	GRHPR	rs10973326	0.0456	0.1320

11	RPUSD4	rs601702	0.0115	0.7258	4	LIAS	rs10016760	0.0059	0.669	cg25669547	10	PRDX3	rs10886413	0.0482	0.9660
12	TXNRD1	rs11111979	0.0067	0.1326	6	LYRM4	rs452908	0.0275	0.611	cg00563771	12	NT5DC3	rs954921	0.0372	0.0823
15	LACTB	rs2729779	0.0052	0.1275	6	MOCS1	rs2504080	0.0043	0.911	cg10871120	19	ECH1	rs2229259	0.0030	0.6360
22	SNAP29	rs5759762	0.0421	0.7399	4	QDPR	rs34914923	0.0073	0.233	cg08808571					
22	SERHL2	rs137112	0.0303	0.5571	4	QDPR	rs34914923	0.0071	0.258	cg26689483					
					7	TBRG4	rs7788562	0.0403	0.995	cg07992143					
					16	TRAP1	rs2791	0.0275	0.607	cg02264195					
					12	TXNRD1	rs1186327	0.0023	0.193	cg25684105					
					22	TXNRD2	rs34606353	0.0457	0.966	cg11182965					
					1	VPS13D	rs74819464	0.0159	0.498	cg09686497					
					1	VPS13D	rs17423806	0.0201	0.317	cg24602020					

QTL, quantitative trait loci

Summary of colocalization results for eQTL, mQTL, and pQTL

Colocalization analysis using eQTL, mQTL, and pQTL data showed no evidence of shared causal variants with TB susceptibility, as indicated by posterior probability hypothesis 4 (PP.H4) values below 0.8 for all analyzed loci (Table S3).

Table S3. Summary of colocalization results for eQTL, mQTL, and pQTL

eQTL		pQTL		mQTL	
**PP.H4**	**gene**	**PP.H4**	**gene**	**PP.H4**	**gene**
0.01712	SNAP29	0.0324	QDPR	0.28158	QDPR
0.30015	TXNRD1	0.01043	PAICS	0.13564	TXNRD1
0.02466	SERHL2	0.00196	ECH1	0.02013	LIAS
0.01702	FIS1	0.00559	NT5DC3	0.05288	TBRG4
0.01978	LACTB	0.01559	FIS1	0.02597	TXNRD2
0.01919	AK3	0.02156	GRHPR	0.03207	GUK1
0.16967	RPUSD4	0.01518	PRKN	0.04282	COMT
0.07392	SFXN4	0.01064	PRDX3	0.01915	ACSF3
0.01889	CRYZ			0.01469	LYRM4
0.11865	PDHB			0.05797	TRAP1
				0.2663	VPS13D
				0.05115	MOCS1

QTL, quantitative trait loci

Tissue-Specific Validation of Mitochondrial Feature Genes

To determine tissue-specific involvement, expression levels of the identified feature genes were validated using GTEx data. We validated tissue-specific expression using GTEx data, and no specific tissue association was observed for PDHB, LYRM4, AK3, and ACSF3, indicating their broad contribution to TB susceptibility rather than tissue-specific involvement (Table S4, Figure S2).

Table S4 Tissue-specific validation of model genes using GTEx data.

**Chr**	**Gene**	**SNP**	**p_SMR**	**p_HEIDI**
3	PDHB	rs57621305	0.833356	0.088449
6	LYRM4	rs466837	0.134604	0.787986
9	AK3	rs79965806	0.852214	0.939737
16	ACSF3	rs28542844	0.090938	0.077112

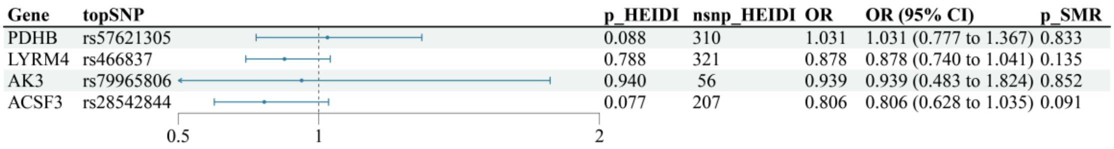

Figure S2: Forest plot illustrating tissue-specific validation outcomes for mitochondrial feature genes.
